# Genetic Diversity of *Staphylococcus aureus* in Buruli Ulcer

**DOI:** 10.1371/journal.pntd.0003421

**Published:** 2015-02-06

**Authors:** Nana Ama Amissah, Corinna Glasner, Anthony Ablordey, Caitlin S. Tetteh, Nana Konama Kotey, Isaac Prah, Tjip S. van der Werf, John W. Rossen, Jan Maarten van Dijl, Ymkje Stienstra

**Affiliations:** 1 Department of Internal Medicine, University of Groningen, University Medical Center Groningen, Groningen, The Netherlands; 2 Department of Bacteriology, Noguchi Memorial Institute for Medical Research, University of Ghana, Legon, Ghana; 3 Department of Medical Microbiology, University of Groningen, University Medical Center Groningen, Groningen, The Netherlands; 4 Pakro Health Centre, Ghana Health Service, Pakro, Ghana; Kwame Nkrumah University of Science and Technology (KNUST) School of Medical Sciences, GHANA

## Abstract

**Background:**

Buruli ulcer (BU) is a necrotizing skin disease caused by Mycobacterium ulcerans. Previous studies have shown that wounds of BU patients are colonized with M. ulcerans and several other microorganisms, including *Staphylococcus aureus*, which may interfere with wound healing. The present study was therefore aimed at investigating the diversity and topography of *S. aureus* colonizing BU patients during treatment.

**Methodology:**

We investigated the presence, diversity, and spatio-temporal distribution of *S. aureus* in 30 confirmed BU patients from Ghana during treatment. *S. aureus* was isolated from nose and wound swabs, and by replica plating of wound dressings collected bi-weekly from patients. *S. aureus* isolates were characterized by multiple-locus variable number tandem repeat fingerprinting (MLVF) and spa-typing, and antibiotic susceptibility was tested.

**Principal Findings:**

Nineteen (63%) of the 30 BU patients tested positive for *S. aureus* at least once during the sampling period, yielding 407 *S. aureus* isolates. Detailed analysis of 91 isolates grouped these isolates into 13 MLVF clusters and 13 spa-types. Five (26%) *S. aureus*-positive BU patients carried the same *S. aureus* genotype in their anterior nares and wounds. *S. aureus* isolates from the wounds of seven (37%) patients were distributed over two different MLVF clusters. Wounds of three (16%) patients were colonized with isolates belonging to two different genotypes at the same time, and five (26%) patients were colonized with different *S. aureus* types over time. Five (17%) of the 30 included BU patients tested positive for methicillin-resistant *S. aureus* (MRSA).

**Conclusion/Significance:**

The present study showed that the wounds of many BU patients were contaminated with *S. aureus*, and that many BU patients from the different communities carried the same *S. aureus* genotype during treatment. This calls for improved wound care and hygiene.

## Author Summary

Buruli ulcer (BU) is a disease of the skin and soft tissue caused by *Mycobacterium ulcerans*. The resulting skin lesions provide a niche for survival of other microorganisms such as *Staphylococcus aureus*, which may cause delayed wound healing. This study investigated the presence, diversity, and spatio-temporal distribution of *S. aureus* in BU patients from Ghana during treatment by isolating the bacteria from nose and wound swabs or wound dressings. *S. aureus* isolates were subsequently characterized by two complementary DNA typing approaches. This showed that 19 (63%) of the 30 investigated BU patients carried *S. aureus*. Five (26%) of these 19 BU patients carried the same *S. aureus* type in their anterior nares and wounds. Seven (37%) patients carried the same *S. aureus* type in their wounds, which is indicative of transmission. Three of them (16%) carried at least two different *S. aureus* types at the same time in their wounds, while five (26%) carried different *S. aureus* types over time. Notably, five (17%) BU patients tested positive for methicillin-resistant *S. aureus* (MRSA). These findings imply that the spatio-temporal diversity of *S. aureus* in BU is most likely related to factors such as antibiotic pressure, and insufficient wound care and hygiene.

## Introduction

Buruli ulcer (BU) is a neglected necrotizing skin disease caused by *Mycobacterium ulcerans*, emerging mainly in West Africa with Benin, Côte d’Ivoire and Ghana bearing the highest burden of disease [1]. The disease usually starts as a painless nodule, plaque, oedema or papule and progresses to form large ulcers when left untreated. The pathology of BU is strongly associated with the production of mycolactone [2], an immunomodulatory macrolide toxin that causes tissue necrosis [3]. The regimen for treatment of BU disease is streptomycin and rifampicin for 2 months [4–6]. Wound care is an important aspect of treatment but frequently facilities and knowledge on appropriate wound care are missing [7]. BU often results in disfiguring complications, such as contractures and sometimes amputations. More than half of the patients have permanent limitations in the performance of daily activities due to seeking treatment at a later stage [8, 9]. Wounds of most BU patients are colonized with several other microorganisms in addition to *M. ulcerans* [10, 11]. Although risk factors for bacterial wound colonization have not been thoroughly studied to date, delayed treatment and insufficient wound management might contribute to colonization and prolonged wound healing. Until now there are only two studies that describe the microorganisms colonizing the wounds of BU patients cultured from superficial swabs, indicating the presence of *Pseudomonas aeruginosa*, *Proteus mirabilis*, *Enterobacteriaceae*, Group A, B and C *Streptococcus*, and *Staphylococcus* spp., including *Staphylococcus epidermidis* and *Staphylococcus aureus*. Notably, between 33% [11] and 38% [10] of the detected *S. aureus* isolates were methicillin-resistant *S. aureus* (MRSA).

*S. aureus* is usually a harmless commensal, carried by about 20–30% of the general population [12, 13]. However, it can transform into a dangerous pathogen causing a wide range of infections in both community and hospital settings. These infectious diseases range from relatively mild skin infections, such as boils and abscesses, to life-threatening conditions such as pneumonia, bacteremia and endocarditis [14, 15]. Nasal colonization with *S. aureus* has been associated with delayed wound healing and prolonged length of stay at burn centres [16] and previous studies have shown a significant risk for the development of autologous wound infections by nasal carriers [17, 18]. In BU patients, mycolactone production limits the primary immune responses and recruitment of inflammatory cells to the site of infection. The mycolactone can thus act as an immunosuppressive agent [19] that predisposes wounds to bacterial colonization and infections.

In this study, we aimed at investigating the diversity and topography of *S. aureus* colonizing BU patients from Ghana during treatment. *S. aureus* isolates cultured from nose and wound swabs, as well as wound dressings were typed by multiple-locus variable number tandem repeat fingerprinting (MLVF) and *spa*-typing. Our results show that wounds of three (16%) *S. aureus*-carrying BU patients were colonized with two different genotypes of *S. aureus*, which were sometimes found in close proximity to each other. Importantly, 28 (31%) isolates from patients, who came from different communities but visited the same health centre for treatment, were grouped into the same MLVF clusters and harboured related *spa*-types, showing the high genetic relatedness of *S. aureus* isolates colonizing BU patients.

## Materials and Methods

### Ethical approval

The ethical committee of the Noguchi Memorial Institute for Medical Research (NMIMR) (FEDERAL WIDE ASSURANCE FWA 00001824) approved the use of clinical samples for this investigation. Samples were collected upon written informed consent from all patients.

### Confirmation of BU cases

Sampling was done at the Pakro Health Centre, in the Eastern region of Ghana. Patients with suspected BU from different communities reported to the health centre for diagnosis. Using the BU 01.N form (www.who.int/buruli/control/ENG_BU_01_N), information such as the age, place of residence, size of lesion (categories: I ≤ 5 cm, II 5–15 cm and III ≥ 15 cm or at critical sites such as the eye and genitals) was obtained before sampling for diagnosis. For the diagnosis of BU, wound swabs from ulcers were collected from patients and kept in 15 ml Falcon tubes containing 2 ml transport medium (Middlebrook 7H9 supplemented with polymyxin, amphotericin B, nalidixic acid, trimethoprim and azlocillin [PANTA]). DNA was extracted from the samples using the modified Boom method [20]. IS*2404* nested PCR was performed as described previously [21]. Patients whose samples tested positive for the presence of the IS*2404* target were confirmed to have BU. Patients visited the health centre daily for antibiotic therapy with rifampicin and streptomycin for two months, and wound management twice a week until their wounds healed.

### Cultivation of *S. aureus*

Nose and wound swabs of 30 PCR-confirmed BU patients were collected bi-weekly from December 2012 until July 2013 (sampling time points designated as: t1 to t13) or until their wounds healed. The patient material was transported to the NMIMR the same day for culture. Swabs were streaked on cysteine lactose electrolyte-deficient (CLED) agar and incubated at 37°C for ~24 h. *S. aureus* was subcultured on blood agar (BA) plates containing 5% sheep blood and incubated overnight at 37°C. In addition, wound dressings that had covered the wounds of patients for a maximum of three days were collected. The topography of *S. aureus* in the wounds was determined by replica plating the wound dressing on CLED agar and incubation of the plates at 37°C for ~24 h. Initially, between 50 and 82 potential *S. aureus* colonies per patient were selected from the wound replica plates and subcultured on CLED agar until colonies were pure. This number was later reduced to 2 to 10 colonies per patient; the respective colonies were selected based on morphology, size and color/pigmentation. In comparison with our initial sample size, this convenience sample was sufficient to pick up >95% of the *S. aureus* types present in each lesion. Identification of *S. aureus* was confirmed using the Pastorex Staph Plus test (Bio-Rad, Marnes-la-Coquette, France). *S. aureus* isolates were subsequently transported to the University Medical Center Groningen (UMCG) on tryptic soy agar containing 5% sheep blood, subcultured on BA, and stored in 17% glycerol at -80°C for further analyses.

### Identification of other microorganisms in the wounds of BU patients

Colonies from the replica plates that did not phenotypically resemble *S. aureus* were identified using matrix-assisted laser desorption ionization-time of flight mass spectrometry (MALDI-TOF MS) with a microflex LT Biotyper (Bruker Daltonics, Bremen, Germany) according to manufacturer’s instructions. Briefly, different colonies were applied onto the MALDI-TOF MS target and overlaid with 1 μl matrix solution, which is a saturated solution of α-cyano-4-hydroxycinnamic acid in 50% acetonitrile and 2.5% trifluoroacetic acid. The target was then air-dried at room temperature and analyzed in the Biotyper. A log score is calculated by the Biotyper software by comparing data of the unknown organism with data of reference organisms for identification of the unknown organism (log score ≥ 2—reliable species identification (ID); log score > 1.7 < 2 reliable genus ID; log score < 1.7 no reliable ID).

### DNA extraction

Total DNA was prepared from *S. aureus* colonies taken from BA plates as described by Glasner *et al*. [22]. Briefly, 2 to 3 colonies were resuspended in 500 μl of Tris-EDTA (TE) buffer (pH 8.0) containing zirconia/silica beads with a diameter of 100 μm in 1.5 ml bead-beating tubes with screw caps. The cells were disrupted using a Precellys bead beater (Bertin Technologies, Saint Quentin en Yvelines, France) in 3 pulses of 30 s at a speed of 5,000 rpm with 30 s intervals between the pulses. Samples were subsequently heated to 95°C for 10 min and centrifuged (14,000 rpm) at 4°C for 10 min. A volume of 200 μl of the resulting supernatant fraction was transferred into a fresh tube and stored at -20°C until further use.

### Detection of the accessory gene regulator, *mecA* and Panton-Valentine leukocidin genes

All *S. aureus* isolates were screened for the presence of the *mecA*, accessory gene regulator (*agr*) (types I, II, III and IV) and Panton-Valentine leukocidin (PVL) (*lukS-PV/lukF-PV*) genes by PCR as described previously [23–26].

### MLVF

MLVF was performed using a modified protocol as described by Glasner *et al*. [22] and Sabat *et al*. [27]. Briefly, 1 μl of genomic DNA was subjected to a multiplex PCR targeting seven staphylococcal genes (*sdrC*, *sdrD*, *sdrE*, *clfA*, *clfB*, *sspA* and *spa*). The resulting PCR products were first separated on 2% agarose gels, because this is most economic. Subsequently, MLVF samples from *S. aureus* isolates with clearly different MLVF banding patterns, or the same MLVF banding patterns but different *agr*, *mecA* and PVL profiles were further analyzed with microfluidic DNA 7500 chips using the Bioanalyzer 2100 (Agilent Technologies, Palo Alto, USA). Notably, these chips have a higher resolution than agarose gels, and the recorded electropherograms can be used for automated data processing. To this end, 1 μl of each PCR reaction was loaded onto a DNA 7500 chip. Next, the PCR-amplified DNA fragments were separated by electrophoresis and electropherograms were automatically recorded according to the manufacturer’s instructions. In each Bioanalyzer run, the clinical *S. aureus* isolate M2, isolated at the UMCG [27] was added as a technical control to ensure the reproducibility of the generated data. The data generated with the Bioanalyzer were imported as CSV files into the GelCompar II software (Applied Maths, Kortrijk, Belgium) for analysis. The position tolerance and optimization were set at 0.9% and 0.5% respectively. Using the selected position tolerance, the M2 control isolate for all Bioanalyzer runs showed identical MLVF banding patterns. Pairwise similarity coefficients were calculated using the dice formula and the dendrogram was generated using the unweighted pair group method using average linkages (UPGMA). Identical patterns were designated as the same MLVF subtype. After visual inspection of the MLVF dendrogram, six different cut-off values (66%, 77%, 79%, 81%, 82% and 84%) were chosen for testing the concordance between MLVF and *spa*-typing. The concordance between the two DNA typing methods was calculated with the Ridom EpiCompare software version 1.0 as described previously [27]. The cut-off value with the highest concordance between the two typing methods was used for clustering of the MLVF patterns.

### *spa*-typing

*spa*-typing of the 91 *S. aureus* isolates was performed as previously described by Harmsen *et al*. [28]. DNA sequences were obtained using an ABI Prism 3130 genetic analyser (Applied Biosystems, Foster City, USA). *spa*-types were determined using the Ridom Staph Type software version 2.2.1 (Ridom GmbH, Würzburg, Germany) [28]. The *spa*-types were grouped into *spa*-clonal complexes (*spa*-CCs) with the based upon the repeat pattern (BURP) algorithm utilizing the Ridom Staph Type software. *spa*-types shorter than 4 repeats were excluded from the analysis, and *spa*-types were clustered if the costs were ≤ 4.

### Antibiotic susceptibility testing

Antibiotic susceptibility was determined with the VITEK 2 system (AST-P633, bioMerieux Corporate, Marcy l’Etoile, France) according to the manufacturer’s instructions. The used card contained the following antibiotics: benzylpenicillin, cefoxitin, chloramphenicol, ciprofloxacin, clindamycin, erythromycin, fosfomycin, fusidic acid, gentamicin, kanamycin, linezolid, mupirocin, oxacillin, rifampicin, teicoplanin, tetracycline, tobramycin, trimethoprim/sulfamethoxazole and vancomycin. The VITEK 2 minimum inhibitory concentration (MIC) results were interpreted using the Advanced Expert System following EUCAST guidelines (www.eucast.org).

## Results

**Figure 1:**
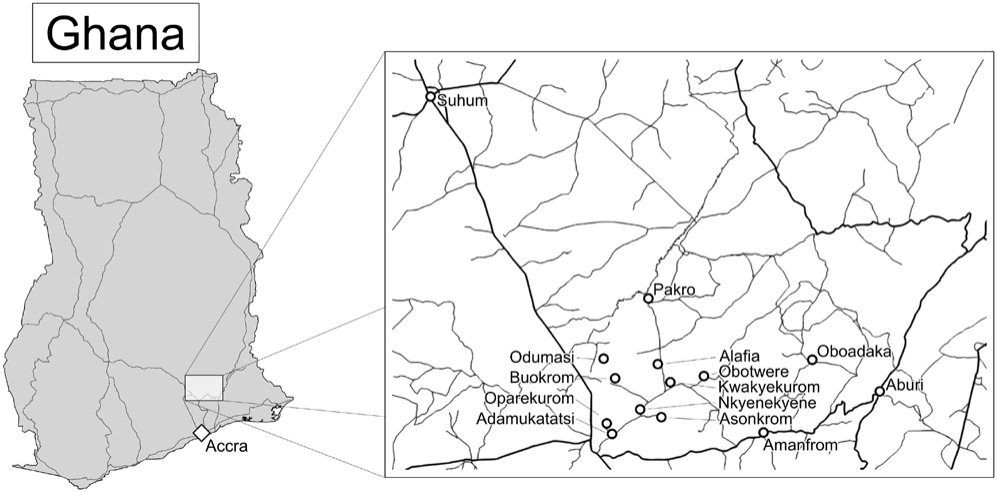
Map of Ghana indicating the region and communities from which the 19 BU patients colonized with *S. aureus* originated.

Thirty of 60 patients receiving treatment at the Pakro Health Centre were confirmed with BU during the time of investigation. These patients had a median age of 44 years, and presented the three disease categories as follows: 30% presented category I lesions, 46.7% category II lesions and 23.3% category III lesions. Clinical signs such as excessive inflammation, exudates and severe pain in the first month of the antimicrobial therapy were observed for the wounds of, respectively, one (3%), two (7%), and 15 (50%) of the 30 BU patients. Samples from 19 (63.3%) of these patients tested positive for *S. aureus* at least once. These 19 patients came from 14 different communities in Ghana (Fig. 1). S1 Fig. displays the frequency of *S. aureus* isolation from the 19 BU patients at different time points (marked t1 to t13). Specifically, *S. aureus* was cultured at least once from the nasal swabs from seven (37%) of these 19 patients, wound swabs from ten (53%) patients and wound dressings from 15 (79%) patients.

### MLVF

**Figure 2:**
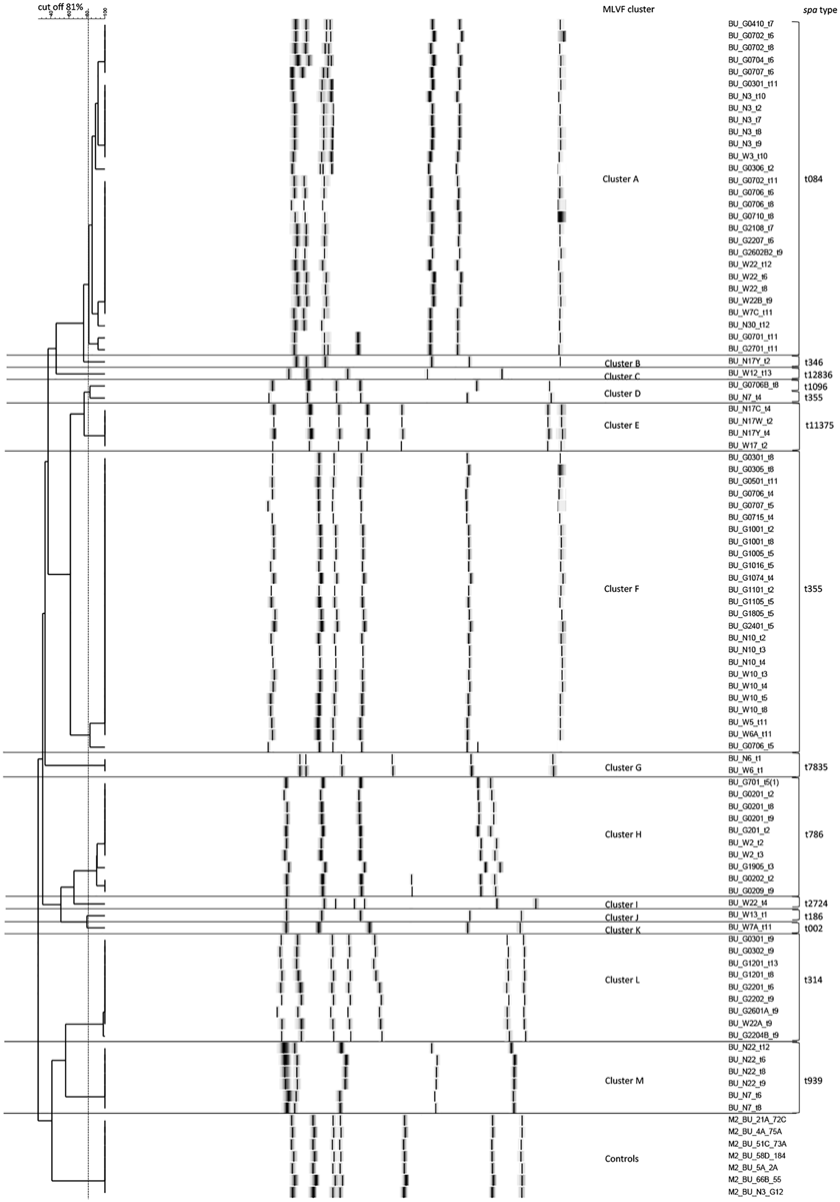
MLVF dendrogram of 91 *S. aureus* isolates from BU patients. The dendrogram was generated using the UPGMA algorithm. Additionally, seven technical controls designated M2 were included in the dendrogram. The respective MLVF clusters and *spa*-types are indicated on the right side of the dendrogram.

The *S. aureus* isolates from the different patient samples were typed by MLVF. Altogether, this involved 407 *S. aureus* isolates from 19 patients. The respective MLVF banding patterns were initially analyzed on agarose gels. Subsequently, 91 *S. aureus* isolates with clearly different MLVF banding patterns, or the same MLVF banding patterns but different *agr*, *mecA* and PVL profiles were selected for a further characterization with microfluidic DNA 7500 chips using the Bioanalyzer 2100. The Bioanalyzer data were then used to generate the dendrogram shown in Fig. 2. Altogether, the MLVF analysis resulted in 24 different banding patterns, of which ten patterns were represented by two or more isolates and 14 patterns by a single isolate. The application of the different cut-off values (66%, 77%, 79%, 81%, 82% and 84%) with the subsequent analyses of the concordance with the identified *spa*-types resulted in Adjusted Rand’s coefficients of 0.952, 0.973, 0.979, 0.980, 0.938 and 0.907, respectively. The cut-off value of 81% yielded the highest concordance (0.980) resulting in 13 clusters of which eight comprised two or more isolates. These 13 clusters were denoted as A (n = 28), B (n = 1), C (n = 1), D (n = 2), E (n = 4), F (n = 25), G (n = 2), H (n = 10), I (n = 1), J (n = 1), K (n = 1), L (n = 9) and M (n = 6). The two major clusters A and F were composed of 28 and 25 isolates respectively, and they were derived from eight patients each (Fig. 2, S1 Table). The clusters A, D, F and G included two or more isolates from the anterior nares and the wounds of individuals patients (five [26%] of 19 BU patients), suggesting autoinoculation from the nose to the wound, or *vice versa*. Furthermore, both clusters H and L include isolates from the wounds of different patients, which is indicative for transmission events between patients. This may also be the case for the MLVF cluster M, which comprised six isolates from the anterior nares of two patients that were not detected in their wounds or wound dressings (Fig. 2, S1 Table).

### *Spa*-typing

**Figure 3:**
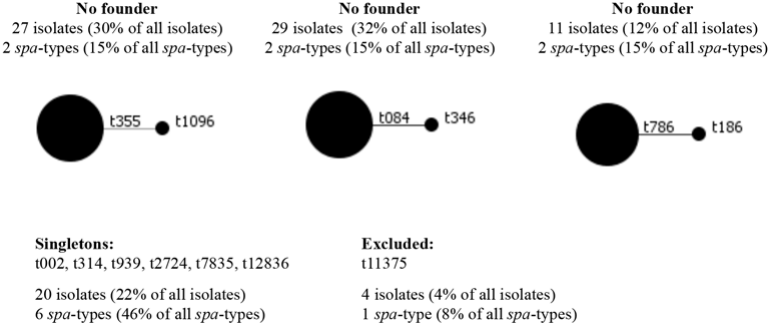
Population structure of the 91 *S. aureus* isolates from BU patients. The population structure was obtained by BURP analysis. In the present *S. aureus* collection, comprising 13 different *spa*-types no *spa*-CCs could be generated. The BURP analysis grouped isolates together into three groups with no founder, comprising 27 (30% of all isolate), 29 (32% of all isolate) and 11 (12% of all isolate) *S. aureus* isolates, respectively. Twenty isolates (22% of all isolates) comprising six *spa*-types (46% of all *spa*-types) were identified as singletons, and four isolates (4% of all isolates) comprising one *spa*-type (t11375, 8% of all *spa*-types) were excluded. The circle size is proportional to the number of isolates in each cluster.

The 91 isolates examined by MLVF on the Bioanalyzer were also characterized by *spa*-typing. This yielded 13 *spa*-types including one new *spa*-type (t12836). Specifically, the numbers of identified repeats ranged between 3 (t11375) and 11 (t084) (Table 1). Seven *spa*-types were represented by two or more isolates and six were represented by single isolates. BURP analysis resulted in no *spa*-CCs in the present collection, but grouped 67 isolates (74% of all investigated isolates) in three groups with no founder (Fig. 3). Twenty isolates (22% of all investigated isolates) comprising six *spa*-types (46% of all *spa*-types) were identified as singletons (Fig. 3), and four isolates (4% of all investigated isolates) comprising one *spa*-type (t11375, 8% of all *spa*-types) were excluded.

**Table 1:** *spa*-types of 91 *S. aureus* isolates from the anterior nares and wounds of 19 BU patients.

**Patient No.**	**Time point of sampling**	***Spa*-type**	***spa* repeats**	**No. of isolates**
2	t2, t3, t8, t9	t786	07–12–21–17–13–34–34–33–34	8
3	t2, t7, t8, t9, t10, t11	t084	07–23–12–34–34–12–12–23–02–12–23	8
	t8	t355	07–56–12–17–16–16–33–31–57–12	2
	t9	t314	08–17–23–18–17	2
4	t7	t084	07–23–12–34–34–12–12–23–02–12–23	1
5	t11	t355	07–56–12–17–16–16–33–31–57–12	2
6	t1	t7835	07–82–21–17–34–34–16–34–33–13	2
	t11	t355	07–56–12–17–16–16–33–31–57–12	1
7	t4	t355	07–56–12–17–16–16–33–31–57–12	5
	t5	t786	07–12–21–17–13–34–34–33–34	1
	t8	t1096	07–56–17–16–16–33–31–57–12	1
	t6, t8	t939	04–16–34–12–34–12	2
	t6, t8, t11	t084	07–23–12–34–34–12–12–23–02–12–23	10
	t11	t002	26–23–17–34–17–20–17–12–17–16	1
10	t2, t3, t4, t5, t8	t355	07–56–12–17–16–16–33–31–57–12	12
11	t2, t5	t355	07–56–12–17–16–16–33–31–57–12	2
12	t8, t13	t314	08–17–23–18–17	2
	t13	t12836	08–13–13–17–34–16–34	1
13	t1	t186	07–12–21–17–13–13–34–34–33–34	1
17	t2	t346	07–23–12–34–12–12–23–02–12–23	1
	t2, t4	t11375	07–56–12	4
18	t5	t355	07–56–12–17–16–16–33–31–57–12	1
19	t3	t786	07–12–21–17–13–34–34–33–34	1
21	t7	t084	07–23–12–34–34–12–12–23–02–12–23	1
22	t4	t2724	26–17–34–20–17–12–17–16	1
	t6, t8, t9, t12	t084	07–23–12–34–34–12–12–23–02–12–23	5
	t6, t9	t314	08–17–23–18–17	4
	t6, t8, t9, t12	t939	04–16–34–12–34–12	4
24	t5	t355	07–56–12–17–16–16–33–31–57–12	1
26	t9	t084	07–23–12–34–34–12–12–23–02–12–23	1
	t9	t314	08–17–23–18–17	1
27	t11	t084	07–23–12–34–34–12–12–23–02–12–23	1
30	t12	t084	07–23–12–34–34–12–12–23–02–12–23	1

### Bacterial topography in wounds of BU patients

To determine the bacterial topography in the wounds of BU patients, used wound dressings were replica plated onto CLED agar plates. This led to the observation of either distinct colonies or confluent growth of *S. aureus* in combination with other microorganisms. Species identification with the microflex LT Biotyper revealed the presence of *Acinetobacter baumannii*, *Corynebacterium striatum*, *Enterococcus casseliflavus*, *Enterococcus faecalis*, *Escherichia coli*, *Klebsiella pneumoniae*, *Pseudomonas aeruginosa*, *S. epidermidis*, *S. haemolyticus*, and/or *S. hominis* in wound dressings from 15 (79%) of the 19 included patients. In fact, highly heterogeneous bacterial wound populations made it impossible to detect *S. aureus* in the case of three BU patients. In the case of one patient (no. 30) no *S. aureus* was identified upon replica plating of the wound dressings, and the only *S. aureus* isolate of this patient was obtained at one time point from the anterior nares (Fig. 2).

**Figure 4:**
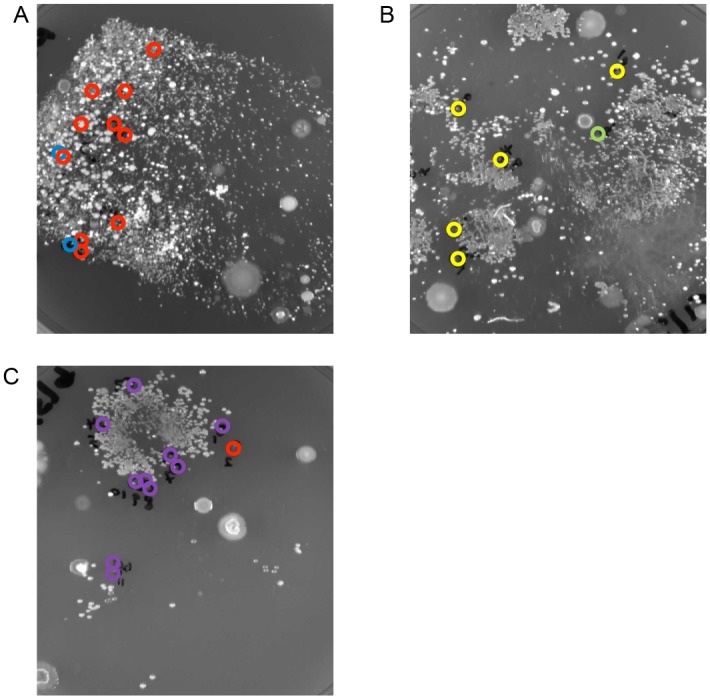
*S. aureus* wound topography in BU patients. Used dressings from wounds of BU patients were replica-plated onto CLED agar plates, and *S. aureus* colonies thus obtained were typed by MLVF. *S. aureus* colonies belonging to different MLVF clusters are shown in different colors: cluster A, red circles; cluster D, blue circles; cluster F, yellow circles; cluster H, green circle and cluster L, purple circles. (A) Replica plate of a wound dressing collected from patient 7 at time point t8 with *S. aureus* colonies belonging to clusters A and D, (B) Replica plate of a wound dressing collected from patient 7 at time t5 with *S. aureus* colonies belonging to clusters F and H. (C) Replica plate of a wound dressing collected from patient 22 at time t6 with *S. aureus* colonies belonging to clusters A and L.

The MLVF typing results of the 49 *S. aureus* isolates obtained from 15 (79%) of the 19 included patients through replica plating of wound dressings were distributed among five different clusters (A, D, F, H and L) (Fig. 2, S1 Table). In this case, the number of isolates selected from a single patient varied from one to six. Interestingly, four of the five clusters, namely A (n = 7), F (n = 7), H (n = 3) and L (n = 4), harboured isolates from patients who reside in different communities (Fig. 2, S1 Table). With regard to the identified *S. aureus* diversity over time in the wounds of the 15 BU patients, different observations were made. In the case of 11 (73%) patients the same *S. aureus* isolates remained detectable over time. For example, patient 2 was colonized at four sampling time points with *S. aureus* isolates that belonged to cluster H, while patients 10 and 11 carried isolates of cluster F for the entire duration of the study (Fig. 2, S1 Table). Three other patients carried *S. aureus* belonging to two MLVF clusters in one wound at one time point. For example, this was the case for patient 22 who was colonized with isolates that grouped in clusters A and L at time point t6 (Fig. 4, S1 Table). For five (33%) patients a temporal change of *S. aureus* genotypes in their wounds was detected as judged by MLVF and *spa*-typing. For example, patient 3 was colonized with *S. aureus* isolates that belonged to cluster A at time t2, cluster F at t8, and cluster L at t9 (S1 Table). Furthermore, patient 12 was colonized with *S. aureus* of cluster L at t8, and at t13 with clusters C and L (S1 Table). Moreover, patient 7 was found to be colonized by *S. aureus* belonging to four MLVF clusters over time. This patient carried *S. aureus* of cluster F at t4, clusters F and H at t5, cluster A at t6, clusters A and D at t8, and cluster A at t11 (Fig. 4, S1 Table).

### Antibiotic resistance pattern and detection of *mecA*, *agr* and PVL genes

All 91 *S. aureus* isolates were tested for their antibiotic susceptibility and the results are presented in Table 2. All 91 isolates were found to be susceptible to clindamycin, fusidic acid, gentamicin, linezolid, mupirocin, tobramycin and vancomycin, while resistance to penicillin was observed in 90 isolates (99%) (S1 Table). Chloramphenicol resistance was observed for 59 (65%) isolates that belonged to the MLVF clusters A, F, G, H, J, L and M. Two isolates (2%) of clusters F and K were resistant to ciprofloxacin. Only one isolate that was grouped in cluster F was resistant to fosfomycin and teicoplanin and displayed an intermediate resistance phenotype to kanamycin. Seven (8%) isolates from the clusters A, F and L were resistant to rifampicin. The latter isolates were obtained from 4 (21%) of the 19 *S. aureus*-positive patients; from three of these patients, rifampicin resistant isolates were obtained 1–5 months after therapy with rifampicin and streptomycin, while rifampicin resistant isolates from one patient were obtained two weeks before the end of the antibiotic therapy. Seventy (77%) isolates belonging to eight clusters (A, D, F, G, H, I, J and L) were resistant to tetracycline. Twenty-seven (30%) isolates belonging to clusters A, B, F, I and L were resistant to trimethoprim/sulfamethoxazole (S1 Table). Twelve (13%) isolates belonging to MLVF clusters F, H, J and K were resistant to cefoxitin and oxacillin, which implies that they are MRSA. Nonetheless, one of these tested negative for the *mecA* and *mecC* genes, suggesting that an as yet unidentified determinant causes the cefoxitin and oxacillin resistance. Interestingly, in wounds of three of the five patients from which oxacillin resistant isolates belonging to MLVF clusters F and H were collected, we also identified methicillin-sensitive *S. aureus* (MSSA) of the same genotype (S1 Table).

**Table 2:** Antibiotic resistances of the 91 *S. aureus* study isolates.

**Antibiotic**	**No. (resistance rate %)**
Ciprofloxacin	2 (2.2)
Chloramphenicol	59 (64.8)
Clindamycin (constitutive)	0 (0)
Erythromycin	2 (2.2)
Fosfomycin	1 (1.1)
Fusidic Acid	0 (0)
Gentamicin	0 (0)
Kanamycin	0 (0)
Linezolid	0 (0)
Mupirocin	0 (0)
Oxacillin	12 (13.1)
Penicillin	90 (98.9)
Rifampicin	7 (7.7)
Teicoplanin	1 (1.1)
Tetracycline	70 (76.9)
Tobramycin	0 (0)
Trimethoprim/sulfamethoxazole	27 (29.7)
Vancomycin	0 (0)

For details on the antibiotic resistances of the respective study isolates, please see S1 Table in the Supporting Information.

Lastly, *agr* typing revealed the presence of all four *agr* types among this collection (type I: 28 (31%) isolates, type II: 26 (29%) isolates, type III: 13 (14%) isolates, and type IV: 17 (19%) isolates). The six (7%) isolates that were grouped in cluster M tested negative for the *agr* gene, and for one isolate (1%) originating from cluster F no *agr* type could be determined as the obtained PCR product did not match with any of the known type-specific PCR products. Six patients were colonized with *S. aureus* isolates grouping into the same MLVF clusters, but belonging to different *agr* groups (S1 Table). Fifty-five (60%) of the 91 isolates obtained from 15 (79%) of the 19 patients over time tested positive for the PVL-encoding genes (S1 Table).

## Discussion

In the present study, we have explored the diversity and topography of *S. aureus* in BU patients as well as the temporal changes in the population of this opportunistic pathogen during patient treatment. To investigate the spatio-temporal distribution of *S. aureus*, MLVF and *spa*-typing were used for the determination of their genotypes.

It has been reported previously that over 80% of the people with skin lesions are nasal *S. aureus* carriers [29, 30]. Furthermore, van der Kooi-Pol *et al*. [31, 32] reported that about 62–75% of the patients with the genetic blistering disease epidermolysis bullosa (EB) were colonized with *S. aureus* in the anterior nares, depending on the absence or presence of chronic wounds, and that 69–92% of these patients carried *S. aureus* in their wounds [32]. In ~60% of the investigated cases, EB patients carried the same type of *S. aureus* in their upper respiratory tract and wounds suggesting a high frequency of auto-inoculation [31]. In the present study, 19 BU patients tested positive for *S. aureus* at least once; 37% of these BU patients were found to contain *S. aureus* at least once in the anterior nares, 79% contained *S. aureus* in their wounds at any one point in time, and 26% contained *S. aureus* with the same genotype in their wounds and nose. Nasal carriage of *S. aureus* in the healthy human population varies between 12–30% [13, 17]. It thus seems that the nasal *S. aureus* carriage rate in BU patients (23% of all 30 included patients) resembles that of the healthy population, and is much lower than in EB patients. On the other hand, the frequency of *S. aureus* wound colonization in all 30 included BU patients (50%) is slightly lower than that of EB patients, while the potential auto-inoculation between the nose and wounds of BU patients (26%) as evidenced by the detection of *S. aureus* isolates with the same MLVF and *spa*-types at both body sites appears substantially lower than observed for EB patients. These differences may relate to the fact that EB patients suffer from recurring wounds right from the moment of birth, while BU patients develop wounds only upon infection with *M. ulcerans*. Accordingly, EB patients are likely to be exposed to *S. aureus* for much longer periods of time than BU patients.

Our present typing analyses show that 98% of the isolates from BU patients that group in the same MLVF cluster have the same *spa*-type (Fig. 2). Only cluster D contained two isolates with different and unrelated *spa*-types, namely t355 and t1096. Within the collection of the 91 investigated isolates, most isolates (58%) were grouped in the MLVF clusters A and F with the respective *spa*-types t084 and t355. In a recent study, these *spa*-types have been reported as the most prevalent types in healthcare institutions in Ghana [33]. Notably, the wounds of the majority of patients followed over time were continuously colonized with the same *S. aureus* MLVF type, and only seven BU patients carried *S. aureus* isolates belonging to different unrelated types. Contrary to these findings, highly variable *S. aureus* types have been isolated from EB and cystic fibrosis patients [34, 35]. Moreover, the genotypes of patient isolates identified in the present study were not specific for particular patients. Instead, we frequently found that different BU patients carried *S. aureus* with the same MLVF and *spa*-types at the same or successive time points (S1 Table). This suggests possible patient transmission events. Since the studied BU patient cohort is composed of patients who all visited the Pakro Health Centre at the same time for antibiotic therapy and wound care treatment, it is conceivable that (indirect) patient-to-patient transmission of *S. aureus* has occurred during this event. This view would be consistent with the finding that *spa*-types t084 and t355 are dominant in healthcare settings in Ghana [33]. On the other hand, we cannot rule out the possibility that transmission events may have occurred in the communities. For the present study we were, unfortunately, unable to obtain samples from the cleansing solutions, surfaces of dressing equipment and the healthcare workers who dressed the wounds of the patients. Samples from the household members of the investigated BU patients could have further informed us on how *S. aureus* and other detected wound-colonizing bacteria are potentially transmitted from family members to patients (i.e. during wound dressing) and subsequently *via* the healthcare setting to other patients. Lack of information on these potential sources makes it difficult to infer the exact source from which these patients were colonized with any certainty. Although the evidence for inter-patient *S. aureus* transmission events that we present here is strong, to obtain final proof for the idea that the *S. aureus* isolates with identical MLVF and *spa*-types from different patients reflect transmission events, it will be necessary to analyze these isolates by a more discriminatory typing method such as whole-genome sequencing (WGS). Data generated by WGS provide a much higher resolution in the investigation of transmission events than any of the more conventional typing methods [36–38].

The analysis of the bacterial topography of the wounds of BU patients showed that wound-colonizing *S. aureus* isolates belonged to the same or two different MLVF clusters at particular time points. A similar investigation on chronic wounds of EB patients also showed the co-existence of distinct *S. aureus* types in close proximity, although in this case the co-existence of up to six distinct *S. aureus* types was observed [35]. Furthermore, our present analysis of the bacterial wound topography of BU patients over time showed that the population of *S. aureus* may shift (Fig. 2, Table 1). This shift may relate to the use of rifampicin and streptomycin for treatment of the *M. ulcerans* infection, but this needs to be further investigated. In any case, only 13% of all the 30 included patients carried rifampicin resistant *S. aureus* isolates in the present study, indicating that the BU treatment might not have a strong effect on the genetic population structure of *S. aureus* in the wounds.

A total of 99%, 77% and 65% of the collected *S. aureus* isolates were resistant to penicillin, tetracycline and chloramphenicol, respectively. This is not surprising as these antibiotics are frequently prescribed in healthcare institutions or readily accessible in pharmacies in Ghana. Similarly, a high resistance to penicillin (73.7–100%) [39, 40] and tetracycline (21.8–92%) [41, 42] has been reported for MSSA isolates in other African countries, including Algeria, Nigeria, and Sao Tome and Principe. Notably, *S. aureus* isolates that belonged to the same MLVF cluster from individual BU patients included in our present study often presented the same antibiotic resistance pattern. Furthermore, 13% of the 91 selected *S. aureus* isolates were MRSA, one of which was a borderline oxacillin resistant *S. aureus*. These MRSA isolates were obtained from 5 of the 30 included BU patients (17%). This implies that the MRSA carriage rate in the sampled BU patient cohort was lower than the 33% [11] and 38% [10] observed in other cohorts of BU patients. From a clinical point of view, it is relevant to mention that three patients carried MRSA and MSSA at the same time, indicating that MRSA can be missed if only single isolates are tested for resistance. This could then result in an inappropriate antibiotic therapy.

Some BU patients were colonized with *S. aureus* that belonged to the same MLVF cluster but had different *agr* types. The presence of different *agr* types of the same *S. aureus* genotype may play different roles by cross-group interference, which may confer a selective survival advantage for these genotypes over time [43]. *agr*-negative *S. aureus* have been shown to have high colonization potential through the formation of biofilms thereby contributing to persistent infection [44, 45]. MLVF cluster M, comprised isolates that are all *agr*-negative. These isolates were obtained from the anterior nares of two patients suggesting that these individuals may be persistent nasal *S. aureus* carriers. Wound colonization of BU patients with PVL-positive *S. aureus* was observed for 79% patients, which appears in line with previous studies that have associated PVL with skin and soft tissue infections [46, 47]. It is tempting to speculate that this could easily be confused with the commonly observed paradoxical response in BU patients [6], where the size of lesions increases upon effective antibiotic therapy against *M. ulcerans*.

In summary, the present study showed that 26% of the investigated BU patients were colonized with the same *S. aureus* types in the anterior nares and wounds during the time period of investigations. Furthermore, patients were colonized with *S. aureus* types that grouped up to two MLVF clusters at the same time point. Over time, changes in the *S. aureus* wound population were observed. Notably, our data suggest that patient transmission events have occurred since some *S. aureus* MLVF clusters were shared by different patients at overlapping time points. This emphasizes the need for appropriate wound care and hospital hygiene. Since *S. aureus* is in general capable of interfering with wound healing and since several of the identified isolates are resistant to clinically relevant antibiotics, it will be important to further investigate the role of *S. aureus* in wound healing in BU patients and to uncover the reservoirs from which this pathogen is transmitted to this patient group.

## Supporting Information

S1 Table *S. aureus* isolates from BU patients listed in the order of the MLVF dendrogram. (XLSX)

S1 Fig Frequency of *S. aureus* isolation from different BU patients. The different colors represent the types of samples from which *S. aureus* isolates were obtained: yellow—nasal swab, orange—wound swab, red—wound dressing. A white field indicates that the patient did not report to the healthcare centre, and a grey field that *S. aureus* was not detected. The successive time points at which samples were collected at the health centre are marked t1 to t13.
(TIF)

S1 Checklist STROBE checklist of items that should be included in reports of observational studies. (DOCX)
