# Supplementary figures and images for: Genetic Diversity of Staphylococcus aureus in Buruli Ulcer
Source: PLoS Negl Trop Dis. 2015 Feb 6;9(2):e0003421. doi: 10.1371/journal.pntd.0003421 (PMC4319846; doi:10.1371/journal.pntd.0003421)

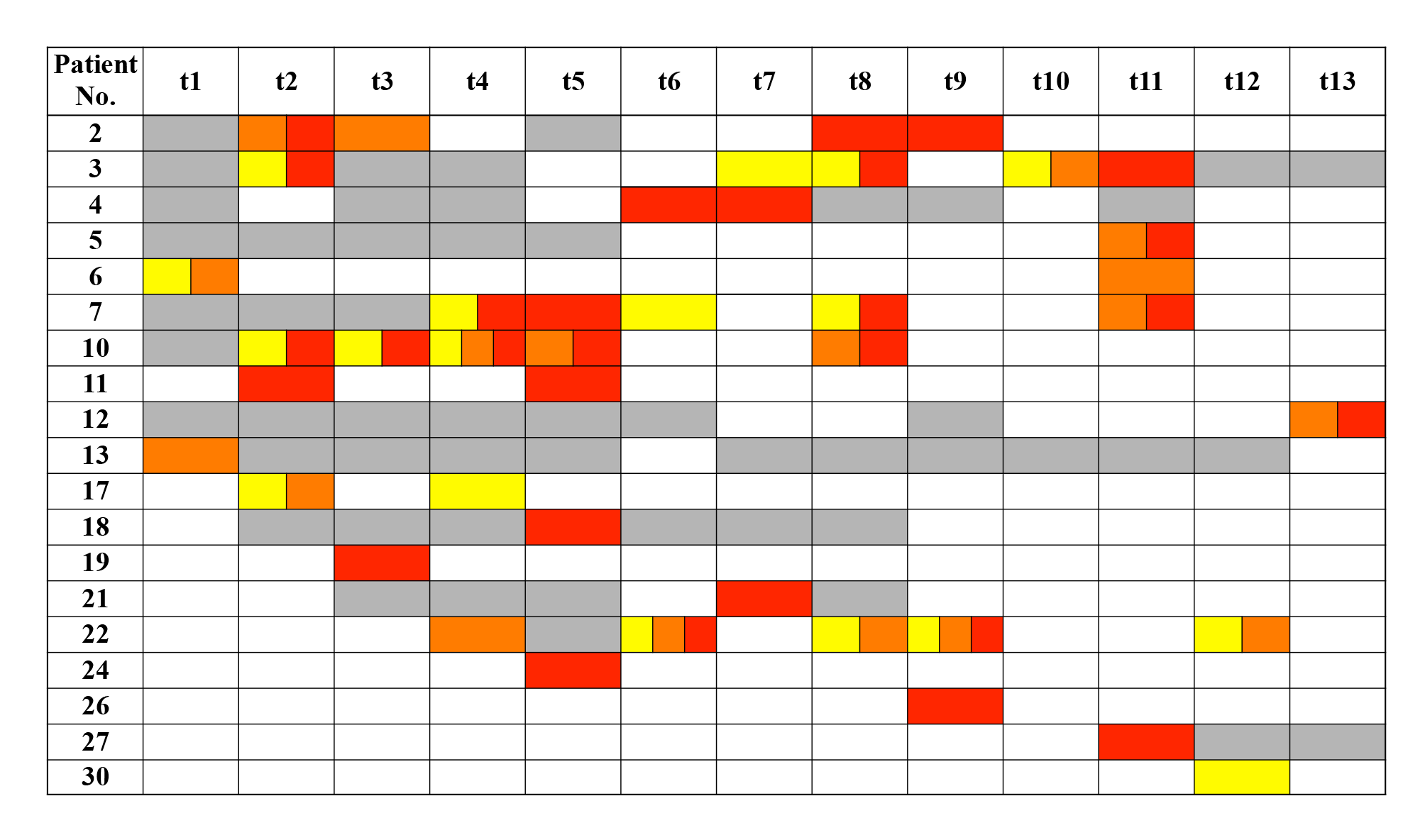

Supplement: S1 Fig — The different colors represent the types of samples from which S. aureus isolates were obtained: yellow—nasal swab, orange—wound swab, red—wound dressing. A white field indicates that the patient did not report to the healthcare centre, and a grey field that S. aureus was not detected. The successive time points at which samples were collected at the health centre are marked t1 to t13. (TIF) [file pntd.0003421.s002.tif]
