# Supplementary material for: Genetic Diversity of Staphylococcus aureus in Buruli Ulcer
Source: PLoS Negl Trop Dis. 2015 Feb 6;9(2):e0003421. doi: 10.1371/journal.pntd.0003421 (PMC4319846; doi:10.1371/journal.pntd.0003421)
Supplement: S1 Checklist — (DOCX) [file pntd.0003421.s003.docx]

| Section | Item No. | Checklist item | Reported on page No. |
| --- | --- | --- | --- |
| Title and abstract | 1a | Indicate the study’s design with a commonly used term in the title or the abstract | 1 |
|  | 1b | Provide in the abstract an informative and balanced summary of what was done and what was found | 2 |
| Introduction |  |  |  |
| Background/rationale | 2 | Explain the scientific background and rationale for the investigation being reported | 4 - 5 |
| Objectives | 3 | State specific objectives, including any prespecified hypotheses | 5 |
| Methods |  |  |  |
| Study design | 4 | Present key elements of study design early in the paper | 5 - 10 |
| Setting | 5 | Describe the setting, locations, and relevant dates, including periods of recruitment, exposure, follow-up, and data collection | 6 |
| Participants | 6a | *Cohort study*—Give the eligibility criteria, and the sources and methods of  selection of participants. Describe methods of follow-up  *Case-control study*—Give the eligibility criteria, and the sources and methods of  case ascertainment and control selection. Give the rationale for the choice of cases  and controls  *Cross-sectional study*—Give the eligibility criteria, and the sources and methods of  selection of participants | 6 - 7 |
|  | 6b | *Cohort study*—For matched studies, give matching criteria and number of  exposed and unexposed  *Case-control study*—For matched studies, give matching criteria and the number of  controls per case | NIL |
| Variables | 7 | Clearly define all outcomes, exposures, predictors, potential confounders, and effect  modifiers. Give diagnostic criteria, if applicable | 6 - 10 |
| Data sources/measurement | 8 | For each variable of interest, give sources of data and details of methods of assessment (measurement). Describe comparability of assessment methods if there is more than one group | 20 – 25, 5 - 10 |
| Bias | 9 | Describe any efforts to address potential sources of bias | Not applicable |
| Study size | 10 | Explain how the study size was arrived at | NIL |
| Quantitative variables | 11 | Explain how quantitative variables were handled in the analyses. If applicable, describe which groupings were chosen and why | NIL |
| Statistical methods | 12a | Describe all statistical methods, including those used to control for confounding | Descriptive statistics |
|  | 12b | Describe any methods used to examine subgroups and interactions | Descriptive statistics |
|  | 12c | Explain how missing data were addressed | NIL |
|  | 12d | *Cohort study*—If applicable, explain how loss to follow-up was addressed  *Case-control study*—If applicable, explain how matching of cases and controls was addressed *Cross-sectional study*—If applicable, describe analytical methods taking account of sampling strategy | NIL |
|  | 12e | Describe any sensitivity analyses | NIL |
| Results |  |  |  |
| Participants | 13a | Report numbers of individuals at each stage of study—eg numbers potentially eligible, examined for eligibility, confirmed eligible, included in the study, completing follow-up, and analyzed | 10 - 15 |
|  | 13b | Give reasons for non-participation at each stage | NIL |
|  | 13c | Consider use of a flow diagram | NIL |
| Descriptive data | 14a | Give characteristics of study participants (eg demographic, clinical, social) and information on exposures and potential confounders | Table S1 |
|  | 14b | Indicate number of participants with missing data for each variable of interest | Figure S1 |
|  | 14c | *Cohort study*—Summarise follow-up time (eg, average and total amount) | NIL |
| Outcome data | 15 | *Cohort study*—Report numbers of outcome events or summary measures over time  *Case-control study—*Report numbers in each exposure category, or summary measures of exposure  *Cross-sectional study—*Report numbers of outcome events or summary measures | NIL |
| Main results | 16a | Give unadjusted estimates and, if applicable, confounder-adjusted estimates and their precision (eg, 95% confidence interval). Make clear which confounders were adjusted for and why they were included | NIL |
|  | 16b | Report category boundaries when continuous variables were categorized | NIL |
|  | 16c | If relevant, consider translating estimates of relative risk into absolute risk for a meaningful time period | NIL |
| Other analyses | 17 | Report other analyses done—eg analyses of subgroups and interactions, and sensitivity analyses | NIL |
| Discussion |  |  |  |
| Key results | 18 | Summarise key results with reference to study objectives | 15 |
| Limitations | 19 | Discuss limitations of the study, taking into account sources of potential bias or imprecision | 17 |
| Interpretation | 20 | Give a cautious overall interpretation of results considering objectives, limitations, multiplicity of analyses, results from similar studies, and other relevant evidence | 15 - 19 |
| Generalisability | 21 | Discuss the generalisability (external validity) of the study results | 19 |
| Other information |  |  |  |
| Funding | 22 | Give the source of funding and the role of the funders for the present study and, if applicable, for the original study on which the present article is based | In the “competing interest &Financial Disclosure” section of the submission form |
